# Population Genetic Structure of Peninsular Malaysia Malay Sub-Ethnic Groups

**DOI:** 10.1371/journal.pone.0018312

**Published:** 2011-04-05

**Authors:** Wan Isa Hatin, Ab Rajab Nur-Shafawati, Mohd-Khairi Zahri, Shuhua Xu, Li Jin, Soon-Guan Tan, Mohammed Rizman-Idid, Bin Alwi Zilfalil, The HUGO Pan-Asian SNP Consortium

**Affiliations:** 1 Human Genome Centre, School of Medical Sciences, Universiti Sains Malaysia, Kelantan, Malaysia; 2 Department of Pediatrics, School of Medical Sciences, Universiti Sains Malaysia, Kelantan, Malaysia; 3 Chinese Academy of Sciences and Max Planck Society (CAS-MPG) Partner Institute for Computational Biology, Shanghai Institutes for Biological Sciences, Chinese Academy of Sciences, Shanghai, China; 4 Institute of Biological Sciences, Faculty of Science, Universiti Malaya, Kuala Lumpur, Malaysia; 5 Department of Cell and Molecular Biology, Faculty of Biotechnology and Biomolecular Sciences, Universiti Putra Malaysia, Selangor, Malaysia; 6 Centre of Research for Computational Sciences and Informatics in Biology, Bioindustry, Environment, Agriculture and Healthcare (CRYSTAL), Faculty of Science, Universiti Malaya, Kuala Lumpur, Malaysia; University of Utah, United States of America

## Abstract

Patterns of modern human population structure are helpful in understanding the history of human migration and admixture. We conducted a study on genetic structure of the Malay population in Malaysia, using 54,794 genome-wide single nucleotide polymorphism genotype data generated in four Malay sub-ethnic groups in peninsular Malaysia (*Melayu Kelantan, Melayu Minang*, *Melayu Jawa* and *Melayu Bugis*). To the best of our knowledge this is the first study conducted on these four Malay sub-ethnic groups and the analysis of genotype data of these four groups were compiled together with 11 other populations' genotype data from Indonesia, China, India, Africa and indigenous populations in Peninsular Malaysia obtained from the Pan-Asian SNP database. The phylogeny of populations showed that all of the four Malay sub-ethnic groups are separated into at least three different clusters. The *Melayu Jawa*, *Melayu Bugis* and *Melayu Minang* have a very close genetic relationship with Indonesian populations indicating a common ancestral history, while the *Melayu Kelantan* formed a distinct group on the tree indicating that they are genetically different from the other Malay sub-ethnic groups. We have detected genetic structuring among the Malay populations and this could possibly be accounted for by their different historical origins. Our results provide information of the genetic differentiation between these populations and a valuable insight into the origins of the Malay sub-ethnic groups in Peninsular Malaysia.

## Introduction

Malays (*Melayu*) are an ethnic group who speak Malayo-Polynesian language which is a member of the Austronesian family [1], [2]. They predominantly inhabit the Malay Peninsula, the east coast of Sumatra and the coast of Borneo [1]. In Peninsular Malaysia, the Malays consist of various sub-ethnic groups which are believed to have different ancestral origins based on their migrations centuries ago [3]. The Malay Peninsula was once a very strategic port and trading centre, connecting Indochina and the Indonesian archipelago [4]. However, migrating populations from surrounding areas has further confounded the investigation of the origin of Malays.

This study aims to investigate whether the different Malay sub-ethnic groups originate from a single population or several populations by exploring the possibility of genetic structuring. The Malay populations in the western (*Melayu Minang*) and southern parts (*Melayu Jawa* and *Melayu Bugis*) of the Peninsular Malaysia were believed to have had more historical and cultural links with the populations from the Indonesian archipelago compared to the Malay populations in north-eastern regions (*Melayu Kelantan*). The existence of Chinese and Indian in the Malay Peninsula with different timelines throughout the centuries brought varying degrees of cultural influences and genetics admixtures to the Malay populations. Substantial influx of Chinese and Indians were started only during the British colonial era to work as laborers in the tin mines and the plantation industry that were mainly concentrated on the west coast of peninsula [5]. Prior to British colonization, Chinese and Indian traders had established strong trading links with the Malay Peninsula. These early contacts did not cause large scale migration but intermarriage and integration between them and the Malays were common [5]. Moreover, the Indians had been conspicuous in the region very much earlier, since the period of the ancient Hindu Malay kingdoms which arose in the 2^nd^ century such as *Chi Tu*, *Gangga Negara, Kadaram* and *Langkasuka* that controlled much of the northern Malay Peninsula [6]. These early Malay states were heavily influenced by concepts of religion, government and arts that were brought by the Indians and traces of this influence can still be found in Malays culture despite the later influence of Islam [6], [7].

In addition, the existence of indigenous *Orang Asli* (aboriginal peoples) populations in the peninsula such as the Negritos (*Jahai* and *Kensui*) and Proto-Malays (*Temuan*) have also raised questions as to whether they are associated with the first wave of human migration from Africa, or belong to the more recent events of Asian human evolution [8], [9]. The Negritos, who speak the Aslian languages which are part of the Austro-Asiatic language family, are of Australo-Malesian affinity and share some common physical features with African pygmy populations, including short stature, woolly hair and dark skin [8], [10]. These nomadic hunter-gatherers are believed to be the earliest settlers and original coastal inhabitants of the Malay Peninsula but the arrival of newcomers forced this group further inland, resulting in them being isolated in forested hilly regions, mainly in northern part of Peninsular Malaysia [10], [11]. Meanwhile, the Proto-Malays who arrived later than Negritos in 2000 BC were seafaring people and settled mostly in the central and southern regions of Peninsular Malaysia [1], [11]. They are Austronesian speakers apart from one tribe, (the *Semelai*) who speak Aslian [9] and embrace people who are similar in appearance to the Malays but of diverse origins, some probably having entered the region by sea in recent centuries whilst others may have been living in the peninsula for thousands of years [9], [10]. In contrast, the present-day Malays of the Malay Peninsula are described as Deutero-Malays, the descendants of the Proto-Malays who had admixed with Siamese, Javanese, Sumatran, Indian, Thai, Arab and Chinese traders [12]. However, according to Fix [10], the original Deutero-Malays migrated from southern China (after the migration of the Proto-Malays) over 1500 years ago and their intermarriages with the Proto-Malays and traders of the ancient trade routes resulted in the diverse recent Deutero-Malay populations that became known currently as the Malays. Hence, Malay population structure analysis would not just provide the information on the genetic differentiation between the populations but would also provide insight into the relationship with the indigenous populations in Peninsular Malaysia.

In this study, we used single nucleotide polymorphisms (SNPs) due to their amenability to high throughput genotyping [13]. SNPs are valuable genetic markers for revealing the evolutionary history of populations [14], [15], [16]. In this analysis more than 54,000 SNPs loci that were shared by 434 individuals were screened to investigate the distribution of genetic variation and population genetic structure of the Malay populations. This number is sufficient to estimate population genetic parameters with statistical confidence [14], [17].

The distance-based approaches that are used in this analysis can detect fine-scale population structure of our studied populations and are not computationally demanding compared to model-based approaches [18]. Inference based on this method does not depend on the modeling assumptions and also requires no special marker selection criteria. In addition, the SNPs analyses with distance-based method are very fast, efficient, robust and able to handle relatively small sample sizes, especially when investigating isolated populations that comprised of few individuals [18], [19], [20]. We implemented these methods to investigate the population genetic structure of four Malay sub-ethnic groups; *Melayu Kelantan*, *Melayu Minang*, *Melayu Jawa*, and *Melayu Bugis* in Peninsular Malaysia. We included the indigenous Proto-Malay and Negrito populations to determine the degree of their genetic relatedness to the Malays.

## Materials and Methods

The population sampling of Peninsular Malaysia Malays were done by following the inclusion and exclusion criteria (Table 1). The SNPs genotype data of 71 unrelated individual of four Malay sub-ethnic groups namely Melayu Kelantan, Melayu Minang, Melayu Jawa and Melayu Bugis were generated by Affymetrix GeneChip Mapping Xba 50 K Array, a microarray chip that enabled researchers to screen over 50,000 SNPs loci in each individual. A total of 58,960 SNPs that have been genotyped for all the sampled individuals were screened under the strict criteria of data quality control. Samples with a call rate below than 90% were excluded from further analysis and after the assessment, 4,166 SNPs (7%) were filtered out (Unmapped to Affymetrix annotation file, chromosome X SNPs and intersection SNPs with downloaded Pan-Asian SNP genotypes), leaving a total of 54,794 autosomal SNPs as the final genotype data for each individual to be used in further analyses.

**Table 1:** List of all studied populations with the location and sample ID.

Population (No. of samples)	Sample ID	Location
Malaysian Melayu^a^:		Peninsular Malaysia:
Kelantan (18)	MY-KN	Kelantan
Minang (20)	MY-MN	Negeri Sembilan
Jawa (19)	MY-JV	Johor
Bugis (14)	MY-BG	Johor
Proto-Malay^b^:		
Temuan (49)	MY-TM	Negeri Sembilan
Negrito^b^:		
Jahai (50)	MY-JH	Perak
Kensui (30)	MY-KS	Kedah
Indonesian^b^:		Indonesia:
Melayu (12)	ID-ML	Sumatra
Jawa (19)	ID-JV	Java Island
Toraja (20)	ID-TR	Sulawesi
Chinese^b^:		China:
Jinuo (29)	CN-JN	Yunnan
Wa (56)	CN-WA	Yunnan
Indian^b^:		India:
Marathi (14)	IN-WL	Maharashtra
Telugu (24)	IN-DR	Andhra Pradesh
African^b^:		Africa:
Yoruba (60)	YRI	Nigeria

a The inclusion criteria are; the sampled individual of a population must be at least three generations of the same population, no parental admixture and communicate daily in the local dialect. The exclusion criteria are those that contradict the inclusion criteria.

b The genotype data that were obtained from the Pan-Asian SNP Consortium database.

The additional 11 populations (Table 1) comprised of Proto-Malays (*Temuan*), Negritos (*Jahai* and *Kensui*), Indonesian Malays (*Melayu*, *Jawa* and *Toraja*), Yunnan Chinese (*Jinuo* and *Wa*), South-West Indians (who speak in *Marathi* and *Telegu* language) and Africans (*Yoruba*) were obtained from the Pan-Asian SNP database [21] (http://www4a.biotec.or.th/PASNP). All of these genotype data were generated from DNA samples that were collected with informed and written consent and approved by local ethics committees (Research and Ethics (Human) Committee, School of Medical Sciences, Universiti Sains Malaysia (USM)) and institutional review board (IRB) of the respective countries.

Allele frequency and genetic distance based on Fixation Index Statistic (Fst) [22] were calculated by PEAS v1.0 [23]. MEGA 4 software [24] and two programs, Neighbor and Consense from PHYLIP 3.67 [25], were implemented to construct the Neighbor Joining tree [26] using all 54,794 autosomal SNPs, shared by 434 individuals from 15 populations. The tree was rooted using *Yoruba* (YRI) as outgroup. Bootstrapping test was performed 1000 times, whereby branches with less than 80% bootstrap values have been dissolved. Multi-dimensional scale analysis was done by SPSS 13 and represented in Euclidean distance three dimension (3D) model.

## Results and Discussion

**Figure 1:**
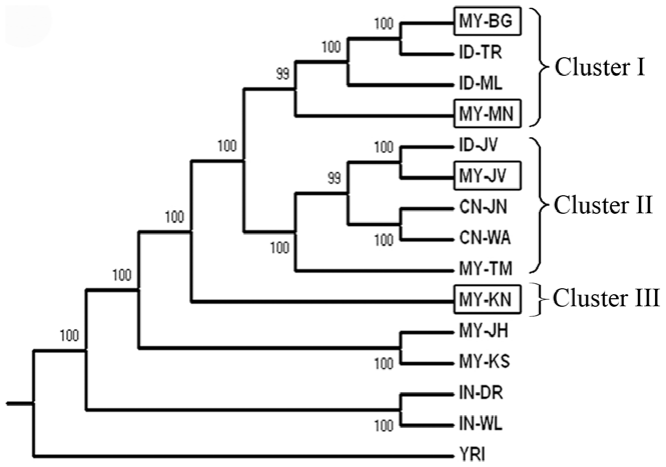
Neighbor-Joining tree of 15 populations based on Fst measurement. In the square boxes are the four studied Malay sub-ethnic groups. Numbers at each branch are represent the percentage value of a thousand bootstrap replications and branches with bootstrap values less than 80% were condensed. The tree suggests a diverse origin of the Malay sub-ethnic groups that forms Cluster I, II and III. Notably, the position of MY-KN in Clade III is the most basal among other studied Malays supported by 100% of bootstrap replicates. There is a distinct genetic difference between the indigenous *Orang Asli* populations; the Negritos is oldest among the peopling groups in Malay Peninsula, whereas the Proto-Malays shared a common ancestry or have had some mixing with the Chinese and Javanese populations.

Fst [22] is a method to show population genetic structure by partitioning genetic variance within populations relative to between populations. The Neighbor Joining tree (Figure 1) based on the genetic distance measure of Fst for the 15 studied populations showed strongly supported nodes (>95% of bootstrap values) and was rooted using *Yoruba* (YRI) as an outgroup. *Yoruba* is an ethnic group from Nigeria and serve as an outgroup to the non-African populations in our study. As the sampling procedure stringently followed the inclusion and exclusion criteria that emphasized the three generations without any different ethnic admixture rule for an individual to be considered as a valid subject for this study, we assumed that there was no recent admixture or gene flow among all the studied populations.

In the Fst tree (Figure 1), each of the Malay sub-ethnic groups in Peninsular Malaysia; *Melayu Kelantan* (MY-KN), *Melayu Minang* (MY-MN), *Melayu Jawa* (MY-JV) and *Melayu Bugis* (MY-BG) is monophyletic, thus establishing there is substructure among Malays. However, the tree does not support the designation of Malays as a monophyletic group since the MY-KN were on a separated clade from other Malay populations, and the MY-JV were more closely related to Proto-Malays and Chinese than other Malays. Generally, the populations were assigned into three different clusters (Cluster I, II, and III) instead of forming a single Malays cluster. Different ancestry across the Malay groups is likely, as they are a paraphyletic class.

In Cluster I, the MY-MN was grouped with Indonesian *Melayu* (ID-ML), whereas MY-BG was grouped with Indonesian *Toraja* (ID-TR). The topology may reflect the migrations of MY-MN and MY-BG to Malay Peninsula from Sumatra and Sulawesi, which are also the geographic origins of ID-ML and ID-TR, respectively. Between these populations, MY-MN appeared as the more basal group than MY-BG, which may suggest populations in Sumatra may have separated earlier than those in Sulawesi.

Cluster II grouped the *Melayu Jawa* (MY-JV) together with Indonesian *Jawa* (ID-JV), which suggest past migration between these populations, or common ancestry. Both populations cluster with the Chinese groups (CN-JN and CN-WA). The Chinese may have had more widespread admixture with the *Jawa* people rather than other Malays, Malaysian and Indonesian in this study. As both Malaysian and Indonesian *Jawa* have very close genetic relationship with the Chinese, it could be postulated that the mixture happened before the migration event of the *Jawa* people to Malay Peninsula around 15^th^ century [27].

In Cluster III, the *Melayu Kelantan* (MY-KN) were basal compared to other Malays on the tree. Interestingly it formed an independent clade and placed outside, rather than within the two mentioned clades. The topology might suggest that MY-KN may have had an ancestry that is more divergent than those of the other Malay populations. This could also be attributed to their geographical location at the northern part of Peninsular Malaysia, which would account for their limited links with populations from the Indonesian archipelago. In contrast, MY-MN, MY-BG and MY-JV, have settled on the western and southern regions of the peninsula in proximity to the Indonesian archipelago.

The other explanation for the paraphyletic nature of the Malay class could be admixture of MY-KN with Indian populations (represented by IN-DR and IN-WL). The influence of Hinduism from India was historically very great and the Malays were largely ‘Indianized’ before they were converted to Islam [7]. Although Hinduism also existed in some of the Indonesian islands (eg. Java Island), it was more predominant among the cultures of populations in mainland Southeast Asia such as Thailand, Cambodia, Myanmar which had more direct contact with the Indian populations [28]. And, the northern part of Peninsular Malaysia had more historical connections with these civilizations [7], [29] since centralization of the ancient Indianized kingdoms had occurred in that region for centuries in the early millennium.

Possible admixture between Malays and Indians was first shown genetically using biochemical markers [30]. Even though the admixture could have occurred during the British colonial period from the 19^th^ to the middle of the 20^th^ century when massive migration of Indian laborers to the west coast of Peninsular Malaysia to work on the railroad and in the rubber and oil palm plantation industries took place [5], we believed that the admixture between MY-KN and Indians was very ancient and had happened during the early existence of the Malays. According to the 2010 Malaysian population census, Malaysia's population is about 28.9 million and the Indian community is the smallest of the three main ethnic groups, comprising 6.8% of the population, with most of them residing in the western and north-western regions of Peninsular Malaysia which are the location of the big cities and large urban areas in the country. In Kelantan state which is the origin of MY-KN, the total population is about 1.67 million and the percentage of the Indian community is only 0.2% of the population. The Indians are not a large component of the Kelantan population either during or after the British colonial era, as it is an agrarian state with lush paddy fields and rustic fishing villages without any plantation industry to attract the Indian immigrants to this north-eastern part of the peninsula [5].

Regarding the phylogenetic affinities of aboriginal peoples in Malaysia, it was revealed that the Proto-Malay, *Temuan* (MY-TM) population was more related to the Chinese and Malays, especially with *Jawa* populations than the Negritos, represented by the *Kensui* (MY-KS) and *Jahai* (MY-JH) populations. This topology is consistent with the fact that the tribal Proto-Malays are believed to have migrated from Yunnan, China about 4,000–6,000 years ago [1]. They were once probably people of coastal Borneo who expanded into Sumatra and the Malay Peninsula as a result of their seafaring way of life [2], [31]. Thus, our results may provide a genetic evidence of the pre-historic migration of Proto-Malays from Yunnan, China.

On the other hand, the Negritos are regarded as the earliest inhabitants of the Malay Peninsula and are probably descended from the Hoabinhians, as their mtDNA variation shows strong evidence for indigenous origins within Malay Peninsula, with time depth of ∼60,000 years ago [9], [32]. However, their origin and the route of their migration to Asia is still a matter of great speculation [33], [34]. Nevertheless, the suggestion of their origin via a southern route of migration from South India is also plausible, as the recent mtDNA studies on ‘relict’ populations of Southeast Asia and the Andaman and Nicobar Islands also point to the human dispersals through the southern exit route [32], [35], [36]. The phylogenetic tree showed a concordance with the facts mentioned above, where the position of the Negritos on the tree were placed at the most basal position among the Malay, Indonesian and Chinese populations. The genetic relationship of the Malays and aboriginal peoples has not only provided some additional insight to the initial peopling in Malay Peninsula, but also may allow one to gauge admixture due to more recent migration.

**Figure 2:**
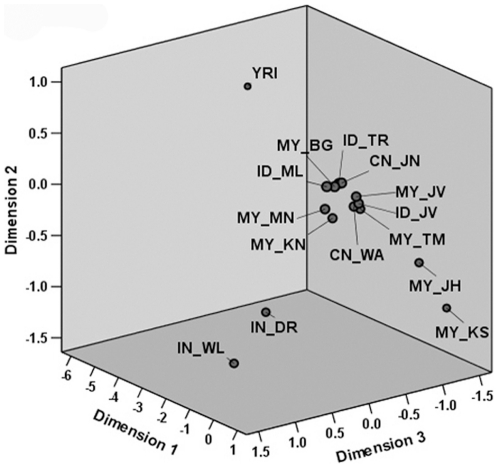
MDS analysis in three dimension model recapitulated the pattern of Fst tree. The 3D MDS showed that all four Malay populations are well separated into three different sub-clusters, although still remained in the same cluster and dimensional platform. They are far separated from three other group populations which are Yoruba, Indian (IN-DR and IN-WL) and Negrito (MY-JH and MY-KS) which are far more diversified than the modern Malays.

The genetic structure of Malays in the Fst tree is recapitulated by multi dimensional scale (MDS) analysis in three dimensions (3D) model as shown in Figure 2. Notably, all four Malay sub-ethnic groups are well separated into three different sub-clusters, although they still remained in the same dimensional platform (dimension 3) indicating an existence of substructure within the Malay population. Malay sub-ethnic groups clustered together with Chinese populations (CN-JN and CN-WA) on the middle area of the dimension 3 platform and far separated from three other group populations which are *Yoruba*, Indians (IN-DR and IN-WL) and Negritos (MY-JH and MY-KS) which are far more diversified than the modern Malays.

### Conclusion

One of the goal of population genetics to understand the nature and extent of human population structure [37]. We have utilized the distance-based clustering method to show the population genetic structure of Malays and the existence of differences among them. The detected substructure of Malays of Peninsular Malaysia indicates the existence of genetic heterogeneity in the population that might relate to the diverse origins and histories. This study has performed investigation of more comprehensive Malay populations that were not included in the report by Pan-Asian SNP research (PASNPI) [21]. The inclusion of the indigenous populations in this study have shown genetic affinities which have not been revealed in previous studies.

Our results illustrate the potential to investigate further the peopling of Peninsular Malaysia by including more ethnic groups not covered in this study. For a culturally mixed country such as Malaysia, where people of various ethnicity practice different lifestyles under many different environments, the knowledge of population genetic substructure is important for proper design of case control association studies and for identifying disease predisposing alleles that may differ across ethnic groups [38]. Only by characterizing genetic variation among individuals and populations, can we gain a better understanding of differential susceptibility to disease, differential response to pharmacological agents and complex interaction of genetic and environmental factors in producing phenotypes [38].

## Supporting Information

Text S1 The participants of the HUGO Pan-Asian SNP Consortium are arranged alphabetically by surname.
(DOC)
