# Supplementary material for: Population Genetic Structure of Peninsular Malaysia Malay Sub-Ethnic Groups
Source: PLoS One. 2011 Apr 5;6(4):e18312. doi: 10.1371/journal.pone.0018312 (PMC3071720; doi:10.1371/journal.pone.0018312)
Supplement: Text S1 — The participants of the HUGO Pan-Asian SNP Consortium are arranged alphabetically by surname. (DOC) [file pone.0018312.s001.doc]

**Text S1. The participants of the HUGO Pan-Asian SNP Consortium are arranged by surname alphabetically in the following;**

Mahmood Ameen Abdulla,^1^ Ikhlak Ahmed,^2^ Anunchai Assawamakin,^3,4^ Jong Bhak,^5^ Samir K. Brahmachari,^2^ Gayvelline C. Calacal,^6^ Amit Chaurasia,^2^ Chien-Hsiun Chen,^7^ Jieming Chen,^8^ Yuan-Tsong Chen,^7^ Jiayou Chu,^9^ Eva Maria C. Cutiongco-de la Paz,^10^ Maria Corazon A. De Ungria,^6^ Frederick C. Delfin,^6^ Juli Edo,^1^ Suthat Fuchareon,^3^ Ho Ghang,^5^ Takashi Gojobori,^11,12^ Junsong Han,^13^ Sheng-Feng Ho,^7^ Boon Peng Hoh,^14^ Wei Huang,^15^ Hidetoshi Inoko,^16^ Pankaj Jha,^2^ Timothy A. Jinam,^1^ Li Jin,^17,38^ Jongsun Jung,^18^ Daoroong Kangwanpong,^19^ Jatupol Kampuansai,^19^ Giulia C. Kennedy,^20,21^ Preeti Khurana,^22^ Hyung-Lae Kim,^18^ Kwangjoong Kim,^18^ Sangsoo Kim,^23^ Woo-Yeon Kim,^5^ Kuchan Kimm,^24^ Ryosuke Kimura,^25^ Tomohiro Koike,^11^ Supasak Kulawonganunchai,^4^ Vikrant Kumar,^8^ Poh San Lai,^26,27^ Jong-Young Lee,^18^ Sunghoon Lee,^5^ Edison T. Liu,^8^ Partha P. Majumder,^28^ Kiran Kumar Mandapati,^22^ Sangkot Marzuki,^29^ Wayne Mitchell,^30,31^ Mitali Mukerji,^2^ Kenji Naritomi,^32^ Chumpol Ngamphiw,^4^ Norio Niikawa,^40^ Nao Nishida,^25^ Bermseok Oh,^18^ Sangho Oh,^5^ Jun Ohashi,^25^ Akira Oka,^16^ Rick Ong,^8^ Carmencita D. Padilla,^10^ Prasit Palittapongarnpim,^33^ Henry B. Perdigon,^6^ Maude Elvira Phipps,^1,34^ Eileen Png,^8^ Yoshiyuki Sakaki,^35^ Jazelyn M. Salvador,^6^ Yuliana Sandraling,^29^ Vinod Scaria,^2^ Mark Seielstad,^8^ Mohd Ros Sidek,^14^ Amit Sinha,^2^ Metawee Srikummool,^19^ Herawati Sudoyo,^29^ Sumio

Sugano,^37^ Helena Suryadi,^29^ Yoshiyuki Suzuki,^11^ Kristina A. Tabbada,^6^ Adrian Tan,^8^ Katsushi Tokunaga,^25^ Sissades Tongsima,^4^ Lilian P. Villamor,^6^ Eric Wang,^20,21^ Ying Wang,^15^ Haifeng Wang,^15^ Jer-Yuarn Wu,^7^ Huasheng Xiao,^13^ Shuhua Xu,^38^ Jin Ok Yang,^5^ Yin Yao Shugart,^39^ Hyang-Sook Yoo,^5^ Wentao Yuan,^15^ Guoping Zhao,^15^ Bin Alwi Zilfalil,^14^ Indian Genome Variation Consortium^2^

^1^Department of Molecular Medicine, Faculty of Medicine, and the Department of Anthropology, Faculty of Arts and Social Sciences, University of Malaya, Kuala Lumpur, 50603, Malaysia. ^2^Institute of Genomics and Integrative Biology, Council for Scientific and Industrial Research, Mall Road, Delhi 110007, India. ^3^Mahidol University, Salaya Campus, 25/25 M. 3, Puttamonthon 4 Road, Puttamonthon, Nakornpathom 73170, Thailand. ^4^Biostatistics and Informatics Laboratory, Genome Institute, National Center for Genetic Engineering and Biotechnology, Thailand Science Park, Pathumtani 12120, Thailand. ^5^Korean BioInformation Center (KOBIC), Korea Research Institute of Bioscience and Biotechnology (KRIBB), 111 Gwahangno, Yuseong-gu, Deajeon 305-806, Korea. ^6^DNA Analysis Laboratory, Natural Sciences Research Institute, University of the Philippines, Diliman, Quezon City 1101, Philippines. ^7^Institute of Biomedical Sciences, Academia Sinica, 128 Sec 2 Academia Road Nangang, Taipei City 115, Taiwan. ^8^Genome Institute of Singapore, 60 Biopolis Street 02-01, 138672, Singapore. ^9^Institute of Medical Biology, Chinese Academy of Medical Science, Kunming, China. ^10^Institute of Human Genetics, National Institutes of Health, University of the Philippines Manila, 625 Pedro Gil Street, Ermita Manila 1000, Philippines. ^11^Center for Information Biology and DNA Data Bank of Japan, National Institute of Genetics, Research Organization of Information and Systems, 1111 Yata, Mishima, Shizuoka 411-8540, Japan. ^12^Biomedicinal Information Research Center, National Institute of Advanced Industrial Science and Technology, 2-42 Aomi, Koto-ku, Tokyo 135-0064, Japan. ^13^National Engineering Center for Biochip at Shanghai, 151 Li Bing Road, Shanghai 201203, China. ^14^Human Genome Center, School of Medical Sciences, Universiti Sains Malaysia, 16150 Kubang Kerian, Kelantan, Malaysia. ^15^MOST-Shanghai Laboratory of Disease and Health Genomics, Chinese National Human Genome Center Shanghai, 250 Bi Bo Road, Shanghai 201203, China. ^16^Department of Molecular Life Science Division of Molecular Medical Science and Molecular Medicine, Tokai University School of Medicine, 143 Shimokasuya, Isehara-A Kanagawa-Pref A259-1193, Japan. ^17^State Key Laboratory of Genetic Engineering and MOE Key Laboratory of Contemporary Anthropology, School of Life Sciences, Fudan University, 220 Handan Road, Shanghai 200433, China. ^18^Korea National Institute of Health, 194, Tongil-Lo, Eunpyung-Gu, Seoul, 122-701, Korea. ^19^Department of Biology, Faculty of Science, Chiang Mai University, 239 Huay Kaew Road, Chiang Mai 50202, Thailand. ^20^Genomics Collaborations, Affymetrix, 3420 Central Expressway, Santa Clara, CA 95051, USA. ^21^Veracyte, 7000 Shoreline Court, Suite 250, South San Francisco, CA 94080, USA. ^22^The Centre for Genomic Applications (an IGIB-IMM Collaboration), 254 Ground Floor, Phase III Okhla Industrial Estate, New Delhi 110020, India. ^23^Soongsil University, Sangdo-5-dong 1-1, Dongjak-gu, Seoul 156-743, Korea. ^24^Eulji University College of Medicine, 143-5 Yong-du-dong Jung-gu, Dae-jeon City 301-832, Korea. ^25^Department of Human Genetics, Graduate School of Medicine, University of Tokyo, 7-3-1 Hongo, Bunkyo-ku, Tokyo 113-0033, Japan. ^26^Department of Paediatrics, Yong Loo Lin School of Medicine, National University of Singapore, National University Hospital, 5 Lower Kent Ridge Road, 119074, Singapore. ^27^Population Genetics Lab, Defence Medical and Environmental Research Institute, DSO National Laboratories, 27 Medical Drive, 117510, Singapore. ^28^Indian Statistical Institute (Kolkata) 203 Barrackpore Trunk Road, Kolkata 700108, India. ^29^Eijkman Institute for Molecular Biology, Jl. Diponegoro 69, Jakarta 10430, Indonesia. ^30^Informatics Experimental Therapeutic Centre, 31 Biopolis Way, 03-01 Nanos, 138669, Singapore. ^31^Division of Information Sciences, School of Computer Engineering, Nanyang Technological University, 50 Nanyang Avenue, 639798, Singapore. ^32^Department of Medical Genetics, University of the Ryukyus Faculty of Medicine, Nishihara, 207 Uehara, Okinawa 903-0215, Japan. ^33^National Science and Technology Development Agency, 111 Thailand Science Park, Pathumtani 12120, Thailand. ^34^Monash University (Sunway Campus), Jalan Lagoon Selatan, 46150 Bandar Sunway, Selangor, Malaysia. ^35^RIKEN Genomic Sciences Center, W502, 1-7-22 Suehiro-cho, Tsurumi-ku, Yokohama 230-0045, Japan. ^36^Department of Biochemistry, University of Hong Kong, 3/F Laboratory Block, Faculty of Medicine Building, 21 Sasson Road, Pokfulam, Hong Kong. ^37^Laboratory of Functional Genomics, Department of Medical Genome Sciences Graduate School of Frontier Sciences, University of Tokyo (Shirokanedai Laboratory), 4-6-1 Shirokanedai, Minato-ku, Tokyo 108-8639, Japan. ^38^Chinese Academy of Sciences-Max Planck Society Partner Institute for Computational Biology, Shanghai Institutes of Biological Sciences, Chinese Academy of Sciences, 320 Yueyang Rd., Shanghai 200031, China. ^39^Genomic Research Branch, National Institute of Mental Health, National Institutes of Health, 6001 Executive Boulevard, Bethesda, MD 20892 USA. ^40^Research Institute of Personalized Health Sciences, Health Sciences University of Hokkaido, Tobetsu 061-0293, Japan.
